# C-reactive protein-to-albumin ratio and six-month mortality in incident hemodialysis patients

**DOI:** 10.1080/0886022X.2023.2182615

**Published:** 2023-03-30

**Authors:** Mariana Sant’Ana, Joana Gameiro, Cláudia Costa, Carolina Branco, Bernardo Marques da Silva, Nadiesda Peres, Ana Cardoso, Ana Mafalda Abrantes, José Agapito Fonseca, Cristina Outerelo, Cristina Resina, José António Lopes

**Affiliations:** a Faculdade de Medicina da Universidade de Lisboa, Universidade de Lisboa, Lisboa, Portugal; b Division of Nephrology and Renal Transplantation, Centro Hospitalar Universitário Lisboa No Lisboa, Portugal; c Division of Nephrology and Renal Transplantation, Centro Hospitalar Universitário Lisboa Norte, Lisboa, Portugal; d Division of Internal Medicine II, Centro Hospitalar Universitário Lisboa Norte, EPE, Lisboa, Portugal; e Division of Internal Medicine II, Centro Hospitalar Universitário Lisboa Norte, Lisboa, Portugal

**Keywords:** Hemodialysis, mortality, inflammation, malnutrition

## Abstract

**Background:**

The first few months of hemodialysis (HD) are associated with a higher risk of mortality. Protein-energy malnutrition is a demonstrated major risk factor for mortality in this population. The C-Reactive Protein to Albumin ratio (CAR) has also been associated with increased mortality risk. The aim of this study was to determine the predictive value of CAR for six-month mortality in incident HD patients.

**Methods:**

Retrospective analysis of incident HD patients between January 2014 and December 2019. CAR was calculated at the start of HD. We analyzed six-month mortality. A Cox regression was performed to predict six-month mortality and the discriminatory ability of CAR was determined using the receiver operating characteristic (ROC) curve.

**Results:**

A total of 787 patients were analyzed (mean age 68.34 ± 15.5 years and 60.6% male). The 6-month mortality was 13.8% (*n* = 109). Patients who died were significantly older (*p* < 0.001), had more cardiovascular disease (*p* = 0.010), had central venous catheter at the start of HD (*p* < 0.001), lower parathyroid hormone (PTH) level (*p* = 0.014) and higher CAR (*p* = 0.015). The AUC for mortality prediction was 0.706 (95% CI (0.65–0.76), *p* < 0.001). The optimal CAR cutoff was **≥**0.5, HR 5.36 (95% CI 3.21–8.96, *p* < 0.001).

**Conclusion:**

We demonstrated that higher CAR was significantly associated with a higher mortality risk in the first six months of HD, highlighting the prognostic importance of malnutrition and inflammation in patients starting chronic HD.

## Introduction

Chronic kidney disease (CKD) has a prevalence ranging from 8 to 16%, which is growing worldwide [1–3]. CKD can progress differently in every patient and only a small part will develop End-Stage Renal Disease (ESRD) [4]. The average number of new ESRD diagnoses worldwide is 144 per million population (pmp) [5] and the prevalence has also been increasing by about 20,000 cases per year [6]. Additionally, ESRD is associated with frequent hospitalizations, higher healthcare costs and mortality [7].

The main causes of mortality in dialysis patients are cardiovascular disease, infectious diseases and malignancy, irrespective of sex, primary kidney disease and age [8]. The most critical phase when it comes to ESRD patients is the start of dialysis [9], not only because of the implications this technique has in patients’ physiology but also due to the loss of residual renal function [10]. Mortality rates in this population are known to be higher in the first few months of dialysis, especially in the first two months [11]. It is estimated that mortality is approximately 7.6% in the first 90 days and 15.2% in the first 180 days in new HD starters [12].

Other than the above-mentioned mortality risk factors, inflammation and malnutrition also exhibit a substantial role in the outcome of incident dialysis patients [13]. Indeed, protein-energy malnutrition is a major risk factor for mortality [14]. There is no unique marker to assess protein-energy malnutrition in CKD patients. Certainly, to assess the nutritional and inflammatory status, a combination of laboratory parameters, body composition, muscle strength measures, and a malnutrition-inflammation score questionnaire, is required [15].

Serum albumin is associated with inflammation and it is an indicator of nutritional status [16]. Studies have suggested that a decrease in serum albumin confers a higher mortality risk in several diseases, namely heart failure, sepsis, and in ESRD [14,17,18]. In addition to albumin, C-Reactive Protein (CRP) is produced in response to infection and is one of the most used markers of inflammation [19,20].

Higher CRP or low serum albumin has been associated with a higher incidence of contrast nephropathy and mortality in previous studies [20–22]. Therefore, the ratio of CRP-to-albumin (CAR) has been used as a surrogate marker of inflammation and nutritional status. An increased CAR at intensive care unit (ICU) admission has recently been associated with increased mortality rates in patients with severe sepsis or septic shock [20]. CAR has also been used as a prognostic marker for inflammation, critically ill and sepsis patients, and terminal cancer patients [23–28], as well as a predictor of mortality in peritoneal dialysis patients (PD) [29].

Given the high mortality rates of ESRD patients, it is important to identify risk factors for mortality. We aimed to determine if the CAR could be used as a predictor of mortality in the first six months in incident hemodialysis patients.

## Materials and methods

We performed a retrospective analysis of adult incident HD patients who started HD between January 2014 and December2019 at the Centro Hospitalar Universitário Lisboa Norte (CHULN) in Lisbon, Portugal.

Incident HD patients were defined as CKD patients who electively started HD, CKD patients who started HD urgently and patients who developed acute kidney injury on CKD and required maintenance HD after hospital discharge. We excluded patients with previous renal replacement therapy (RRT), namely peritoneal dialysis or renal transplant. We also excluded patients lost to follow-up.

### Variables and outcomes

The following variables were collected: demographic characteristics (age, sex, race); comorbidities [Hypertension, Diabetes mellitus, Heart failure, Ischemic cardiomyopathy, chronic obstructive pulmonary disease (COPD), Cerebrovascular disease, Dementia, Cancer, Peripheral artery disease, cardiovascular disease (CVD), and/or chronic liver disease (CLD)]; HD access (type of access); laboratory work-up at start of HD [hemoglobin, serum creatinine, serum ferritin, serum parathyroid hormone (PTH), estimated glomerular filtration rate (eGFR), serum urea, C-reactive protein (CRP), albumin]; viral serologies (Hepatitis B Virus (HBV), Hepatitis C Virus (HCV), Human Immunodeficiency Virus (HIV)).

The primary outcome was mortality within six months of HD initiation. Secondary outcomes were mortality one and two years after the start of HD.

### Definitions

The presence of CKD was defined as an eGFR lower than 60 mL/min/1.73m2. Presence of comorbidities was ascertained from documentation in the individual electronic clinical records. CAR was calculated as C-reactive protein/albumin.

### Statistical methods

Normality of distribution was determined with Kolmogorov–Smirnov test. Continuous variables were compared using Student’s *t*-test and described as the mean ± standard deviation. Categorical variables were compared with the Chi-square test and described as the total number and percentage of each category. All variables were submitted to univariate analysis and variables with a significant statistical difference underwent multivariate analysis using the Cox regression method. Data were described as hazard ratios (HR) with 95% confidence intervals (CI). Statistical significance was established as a *p*-value lower than 0.05.

A receiver operating characteristic (ROC) curve was used to assess the discriminatory ability of albumin, CRP, and CAR to predict six-month mortality. The ROC curves were compared using the *z*-test. A cutoff value for CAR was determined using the Youden’s Index and defined as that with the highest sensitivity and specificity.

A Kaplan–Meier curve was produced to analyze the cumulative survival according to CAR.

Statistical analysis was performed with the statistical software package SPSS for Windows (version 21.0).

## Results

**Figure 1.**
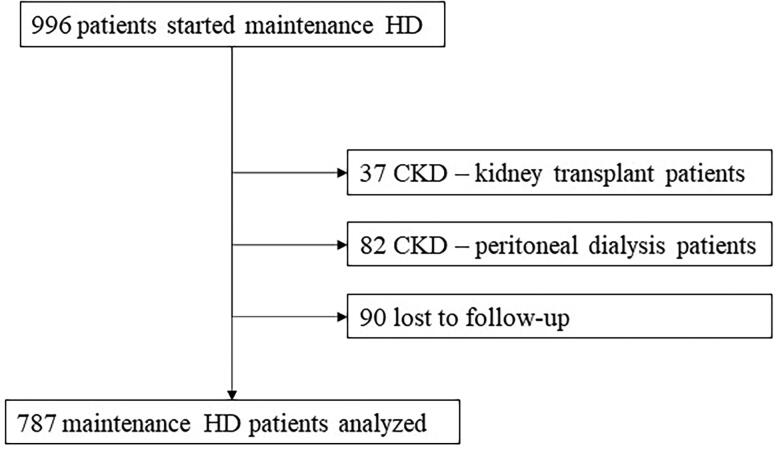
Flow-chart of patient selection.

We analyzed a total of 787 ESRD patients who started maintenance HD at CHULN (Figure 1). The mean age was 68.34 ± 15.5 years and the majority were male (60.6%) and Caucasian (83.9%). Baseline characteristics are described in Table 1.

**Table 1.** Baseline characteristics and according to six-month mortality.

Characteristics	Total (*n* = 787)	Six-month mortality (*n* = 109)	Surival (*n* = 678)	*p*-value
Age (year)	68.3 ± 15.5	76.5 ± 11.4	67.3 ± 15.5	<0.001
Sex (Male) – *n* (%)	477 (60.6)	65 (59.6)	412 (60.7)	0.822
Race (Caucasian) – *n* (%)	661 (83.9)	99 (90.8)	562 (82.9)	0.036
Comorbidities – *n* (%)				
Hypertension	701 (89.1)	93 (85.3)	608 (89.6)	0.176
Diabetes	359 (45.6)	48 (44.0)	311 (45.9)	0.721
Heart failure	282 (35.8)	57 (52.3)	225 (33.1)	<0.001
Ischemic cardiopathy	186 (23.6)	33 (30.3)	153 (22.6)	0.079
COPD	79 (10.0)	14 (12.8)	65 (9.6)	0.294
Cerebrovascular disease	126 (16.0)	22 (20.1)	104 (15.3)	0.200
Dementia	40 (5.1)	9 (8.3)	31 (4.6)	0.105
Cardiovascular disease	383 (48.7)	71 (65.1)	312 (46.0)	<0.001
Cancer	159 (20.2)	29 (26.6)	130 (19.2)	0.073
Peripheral artery disease	134 (17.0)	25 (22.9)	109 (16.1)	0.077
Chronic liver disease	33 (4.2)	4 (3.7)	29 (4.3)	0.767
Laboratory at HD start				
Hemoglobin (g/dL)	9.7 ± 1.7	9.6 ± 1.6	9.7 ± 1.64	0.554
Serum Creatinine (mg/dL)	6.8 ± 3.1	5.7 ± 2.4	7.0 ± 3.1	<0.001
eGFR (ml/min/1.73m2)	9.3 ± 5.8	11.0 ± 9.0	9.1 ± 5.0	0.002
Albumin (g/dL)	3.4 ± 0.7	3.0 ± 0.7	3.5 ± 0.7	<0.001
PTH (pg/mL)	353.2 ± 188.6	229.4 ± 170.5	365.9 ± 215.8	0.006
Ferritin (ng/mL)	527.4 ± 394.4	544.8 ± 368.7	537.5 ± 359.6	0.972
C-RP (mg/dL)	4.4 ± 2.6	6.9 ± 3.0	4.0 ± 2.5	<0.001
Urea (mg/dL)	196.9 ± 80.8	179.3 ± 73.5	198.2 ± 73.8	0.014
CRP/Alb	1.6 ± 0.7	2.9 ± 1.9	1.4 ± 0.4	<0.001
Central venous catheter at HD start – *n* (%)	486 (61.8)	91 (83.5)	395 (58.3)	<0.001

Acronym: COPD: Chronic Obstructive Pulmonary Disease; PTH: Parathyroid Hormone; C-RP: C Reactive Protein; CRP/Alb: C-reactive protein to albumin ratio.

As for comorbidities, a majority of patients had hypertension (89.1%), and a significant percentage of diabetes (45.6%) and heart failure (35.8%). The majority of patients (*n* = 591, 75.1%) had prior Nephrology follow-ups.

Concerning laboratory assessments at the start of HD, mean hemoglobin was 9.7 ± 1.6 g/dL, eGFR was 9.3 ± 5.8 mL/min/1.73m^2^, serum creatinine was 6.8 ± 3.1 g/dL, CRP was 4.4 ± 2.6 mg/dL, and albumin was 3.4 ± 0.7 g/dL. The mean CAR was 1.8 ± 0.74. The vascular access at HD initiation was a central venous catheter (CVC) in 61.8% of patients.

The six-month mortality after start of HD was 13.8% (*n* = 109), one-year mortality was 17.8% and two-year mortality was 26.6%.

Patients who died within the first 6 months were significantly older (unadjusted hazard ratio (HR) 1.06 (1.04–1.07), *p* < 0.001), more frequently Caucasian (HR 2.04 (1.04–4.04), *p* = 0.040), had more frequently heart failure (HR 2.21 (1.47–3.32), *p* < 0.001), and cardiovascular disease (HR 2.19 (1.44–3.34), *p* < 0.001), and had a CVC at the start of HD (HR 3.62 (2.14–6.14), *p* < 0.001).

Concerning laboratory findings at the beginning of HD, mortality was higher in patients with lower serum creatinine (HR 0.82 (0.74–0.90), *p* < 0.001), lower albumin (HR 0.31 (0.22–0.43), *p* < 0.001), higher CRP (HR 1.05 (1.03–1.08), *p* < 0.001) and higher CAR (HR 1.16 (1.09–1.24), *p* < 0.001) (Table 2).

**Table 2.** Univariate and Multivariate analysis of factors predictive of six-month mortality.

Characteristic	Mortality			
	Unadjusted HR (95% CI)	*p*-value	Adjusted HR (95% CI)	*p*-value
Age	1.06 (1.04–1.07)	<0.001	1.06 (1.03–1.09)	<0.001
Sex (Male)	0.95 (0.63–1.44)	0.822		
Race (Caucasian)	2.043 (1.04–4.04)	0.040	0.66 (0.21–1.52)	0.258
Co-morbidities				
Hypertension	0.67 (0.37–1.20)	0.179		
Diabetes	0.93 (0.62–1.40)	0.721		
Heart failure	2.21 (1.47–3.32)	<0.001		
Peripheral artery disease	1.55 (0.95–2.54)	0.079		
Cerebrovascular disease	1.40 (0.39–2.33)	0.202		
Ischemic Cardiopathy	1.49 (0.94–2.33)	0.080		
Dementia	1.88 (0.87-–4.06)	0.110		
CVD	2.20 (1.44–3.34)	<0.001	2.21 (1.21–4.04)	0.010
COPD	1.39 (0.75–2.58)	0.295		
CLD	0.85 (0.29–2.47)	0.767		
Cancer	1.53 (0.96–2.44)	0.074		
Central venous catheter at HD start	3.62 (2.136–6.142)	<0.001	3.09 (1.58–6.03)	0.001
Laboratory findings				
Hemoglobin	0.96 (0.85–1.09)	0.553		
Serum Creatinine	0.82 (0.74–0.90)	<0.001		
Urea	1.00 (0.993–0.999)	0.013	1.00 (0.996–1.004)	0.895
eGFR	1.05 (1.01–1.08)	0.004	1.03 (0.98–2.00)	0.269
Albumin	0.31 (0.22–0.43)	<0.001		
PTH	1.00 (0.996–0.999)	0.002	1.00 (0.997–1.000)	0.014
Ferritin	1.00 (1.00–1.00)	0.97		
C-RP	1.05 (1.03–1.08)	<0.001		
CAR	1.16 (1.09–1.24)	<0.001	1.13 (1.02–1.24)	0.015
CAR ≥ 0.5	5.36 (3.21–8.96)	<0.001		

Acronym: CVD: Cardiovascular Disease; COPD: Chronic Obstructive Pulmonary Disease; CLD: Chronic Liver Disease; PTH: Parathyroid Hormone; C-RP: C Reactive Protein; CAR: C-reactive protein to albumin ratio.

**Figure 2.**
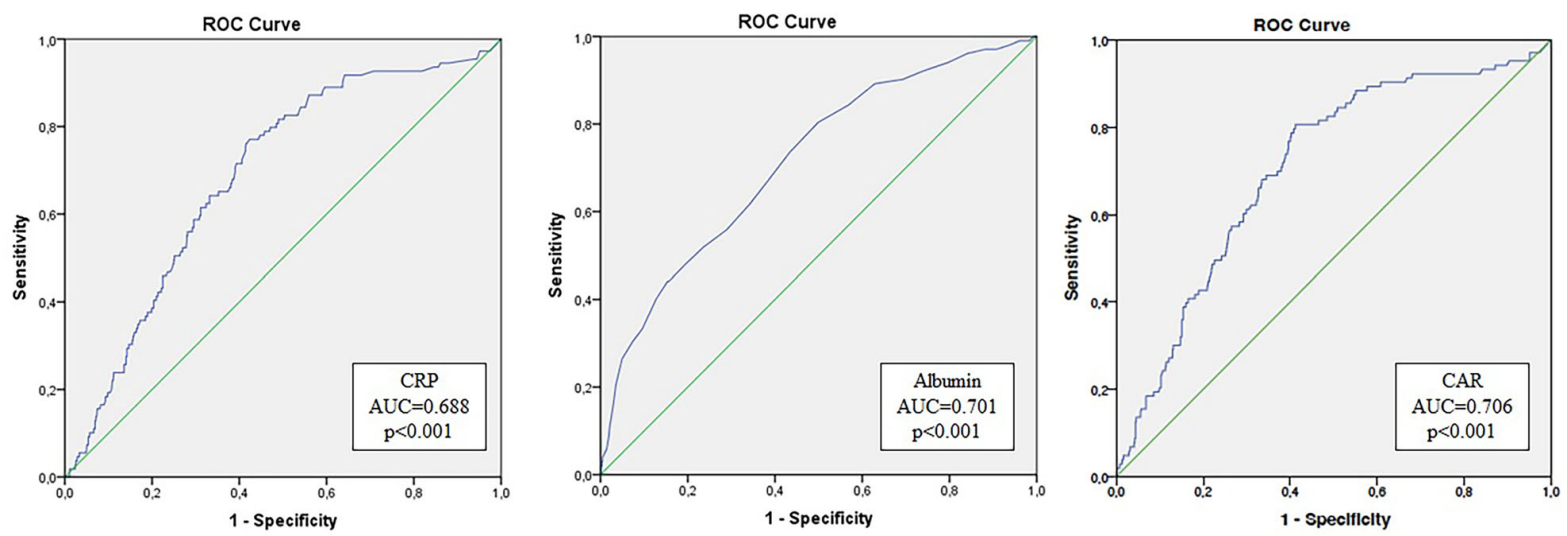
Receiver operating curve of C-reactive protein, albumin, and C-reactive protein-to-albumin ratio to predict six-month mortality.

The AUC for mortality prediction with CRP was of 0.69 (95% CI (0.66–0.77), *p* < 0.001), with albumin, was of 0.70 (95% CI (0.64–0.74), *p* < 0.001), and with CAR was of 0.71 (95% CI (0.65–0.76), *p* < 0.001), z-statistic between the curves was 4.98, *p* < 0.001 (Figure 2).

The optimal cutoff for CAR was assessed to be **≥**0.5, which has a sensitivity of 80.6% and specificity of 56.4%, a positive predictive value of 23.7% and a negative predictive value of 94.5%, meaning that more than 90% of patients with a CAR lower than 0.5 will survive.

Patients with a CAR **≥** 0.5 were older (*p* < 0.001), were more frequently Caucasian (*p* = 0.005), had more frequently cardiovascular disease (*p* < 0.001) and cancer (*p* < 0.001), had lower hemoglobin (*p* < 0.001), lower PTH (*p* = 0.036), and higher ferritin levels (*p* = 0.007). These patients also started HD more frequently using a CVC (*p* < 0.001) (Table 3).

**Table 3.** Characteristics according to CAR ≥ 0.5.

Characteristics	CAR < 0.5 (*n* = 427)	CAR ≥ 0.5 (*n* = 360)	*p*-value
Age (year)	65.0 ± 16.5	72.1 ± 13.6	<0.001
Sex (Male) – *n* (%)	259 (60.7)	219 (60.8)	0.877
Race (Caucasian) – *n* (%)	342 (80.1)	318 (88.3)	0.005
Comorbidities – *n* (%)			
Hypertension	378 (88.5)	327 (90.8)	0.556
Diabetes	202 (47.3)	151 (41.9)	0.097
Heart failure	124 (29.0)	152 (0.42)	<0.001
Ischemic cardiopathy	86 (20.1)	93 (25.8)	0.070
COPD	29 (6.8)	54 (15.0)	<0.001
Cerebrovascular disease	59 (13.8)	64 (17.8)	0.146
Dementia	18 (4.2)	23 (6.4)	0.187
Cardiovascular disease	172 (40.3)	201 (55.8)	<0.001
Cancer	65 (15.2)	93 (25.8)	<0.001
Peripheral artery disease	58 (13.6)	73 (20.3)	0.015
Chronic liver disease	14 (3.3)	18 (5.0)	0.233
Laboratory at HD start			
Hemoglobin (g/dL)	10.0 ± 1.7	9.4 ± 1.5	<0.001
Serum Creatinine (mg/dL)	6.9 ± 2.7	6.9 ± 3.7	0.926
eGFR (ml/min/1.73m2)	9.0 ± 4.3	9.8 ± 7.2	0.066
Albumin (g/dL)	3.7 ± 0.6	3.1 ± 0.6	<0.001
PTH (pg/mL)	376.4 ± 100.1	312.3 ± 143.2	0.036
Ferritin (ng/mL)	371.6 ± 199.8	745.2 ± 563.1	0.007
C-RP (mg/dL)	0.6 ± 0.6	8.9 ± 1.8	<0.001
Urea (mg/dL)	200.5 ± 79.0	193.4 ± 85.9	0.231
Central venous catheter at HD start – *n* (%)	231 (54.1)	258 (71.7)	<0.001
Six-month mortality	20 (4.7)	83 (23.1)	<0.001

**Figure 3.**
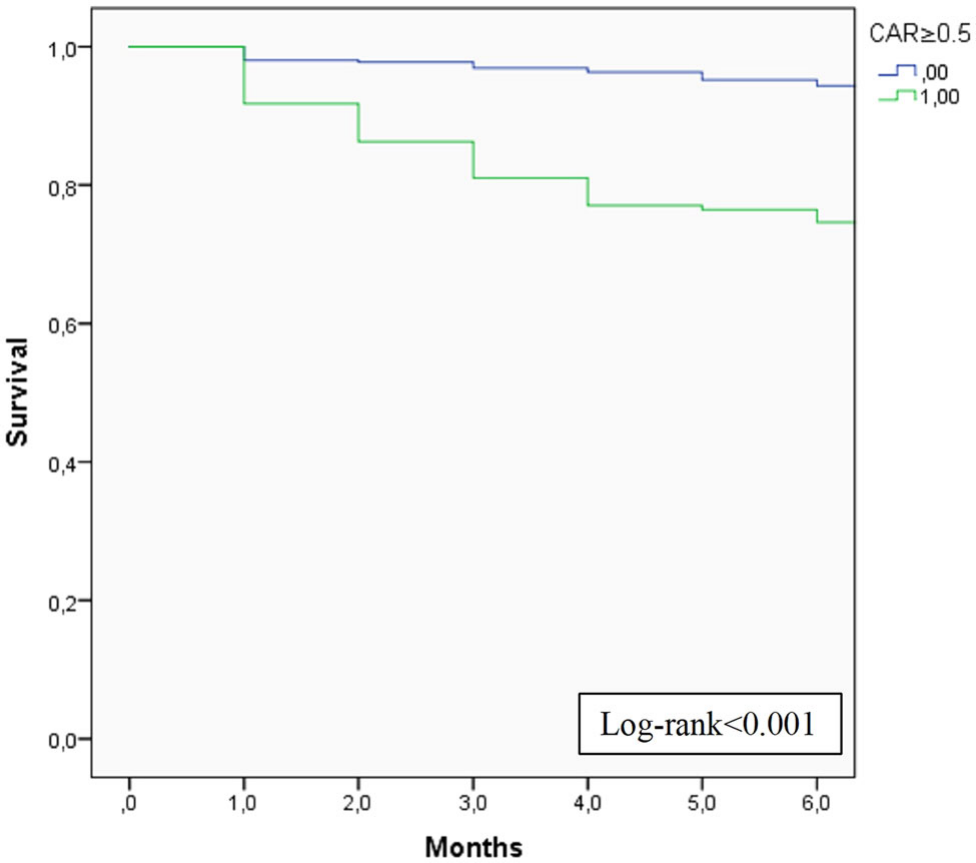
Cumulative survival of patients who started maintenance HD according to CAR ≥ 0.5.

On a multivariate analysis, older age (adjusted hazard ratio (aHR) 1.06 (1.03–1.09), *p* < 0.001), cardiovascular disease (aHR 2.21 (1.21–4.04), *p* = 0.010), CVC at start of HD (aHR 3.09 (1.58-6.03), *p* < 0.001), lower PTH (aHR 0.998 (0.997–1.000), *p* = 0.014) and higher CAR (aHR 1.13 (1.02–1.24), *p* = 0.015) were independent predictors of early mortality. On the multivariate analysis, a CAR ≥ 0.5 had a hazard ratio of 5.362 (95% CI 3.21–8.96, *p* < 0.001) for six-month mortality. The cumulative survival according to CAR ≥ 0.5 is displayed in Figure 3 (log-rank <0.001).

In a further exploratory analysis, CAR **≥** 0.5 was also associated with one-year (HR 4.65 (3.06–7.55), *p* < 0.001) and two-year mortality (HR 4.20 (2.92–6.05), *p* < 0.001) of incident HD patients.

## Discussion

To the best of our knowledge, this study is the first to demonstrate the association of a high CAR at HD initiation and increased mortality risk in the first six months after start of HD, highlighting the impact of malnutrition and inflammation on patient outcomes.

According to the USRDS, the mortality rate of HD patients is 166 per 1000 patient-years, mainly due to cardiovascular disease [30]. Mortality in the first year of HD is the highest and lowers during the second year [30]. Cardiovascular mortality within the first four months of HD is significantly higher compared to the general age-matched population [30]. Lukowsky reported a mortality rate of 36% in the first two years, associated with older age, presence of CVC, and prevalence of cardiovascular disease [11]. This is similar to our report. Considering how the first months after starting dialysis are a critical period for cardiovascular complications, clinical vigilance of the most vulnerable patients is vital.

The association between CAR and mortality rate reinforces the impact of malnutrition and inflammation on cardiovascular mortality of ESRD patients. The presence of the association of malnutrition, inflammation, and atherosclerosis in this population correlates with high mortality rate [31].

Up to 50% of HD patients develop protein-energy malnutrition. Malnutrition is multifactorial and may result from a combination of inadequate food intake, dietary restrictions, increased inflammation, oxidative stress, nutrient losses in urine or dialysate, decreased levels of anabolic hormones and increase of catabolic hormones, metabolic acidosis, aging, and physical deconditioning [32].

Inflammation is an important contributor to several complications in kidney disease, for instance, increased protein degradation rate of skeletal muscle, lesser creation of muscle protein, and insulin resistance. Atherosclerosis is a frequent complication in ESRD patients due to inflammation, malnutrition, and increased oxidative stress, which lead to anomalies in lipid and lipoprotein metabolism as well as endothelial cell dysfunction [33].

Keeping in mind that both inflammation and malnutrition have been correlated with higher mortality rates, CAR is a promising marker to evaluate how both malnutrition and inflammation impact the mortality rate in incident HD patients. In this cohort, the combination of CRP and albumin in the ratio improved the discriminative ability and produced a higher AUC for early mortality than CRP or albumin alone.

Indeed, CAR has been already used to assess mortality in other situations. In a retrospective study of 875 critically ill patients, Park et al. concluded that a higher CAR at ICU admission was independently associated with increased 28-day mortality in these patients (HR = 1.68, 95% CI = 1.27–2.21, *p* < 0.001). They also concluded that admission CAR cutoff values of 34.3 were predictive of 28-day mortality, based on the ROC curve (0.594, *p* < 0.001). However, their sensibility (64.2%) and specificity (52.7%) for predicting mortality were not so high in this single-center study [20]. In our cohort, sensibility and specificity were both higher which allows for more accurate identification of patients at risk.

In a retrospective study with 334 septic shock patients, Ranzani et al. concluded that a higher CAR at ICU discharge was independently associated with higher 90-days mortality and demonstrated a lower survival in patients with CAR >2 (log-rank test: *p* = 0.002) [21].

Similarly, Oh et al. performed a study on 547 ICU patients and found that CAR at admission was associated with increased risk of 30-day mortality. Nevertheless, its prognostic value was significantly inferior than APACHE II or Charlson comorbidity index, which has limited the generalization of this marker [25]. Although we did not evaluate the prognostic value of CAR against other markers, the combination of an inflammation and a nutritional marker is expected to result in increased accuracy in predicting mortality.

A high CAR has also been demonstrated as a mortality risk marker in patients with acute pancreatitis and cancer [24,26,34]. Concerning ESRD patients, Liu et al. used the CAR to predict mortality in 758 incident PD patients. On a 27-month follow-up, a higher CAR was associated with increased all-cause mortality (HR 1.93 (1.08–3.46); *p* = 0.027) [29]. Our study was the first to demonstrate this association in incident HD patients, also indicating the importance of this marker in ESRD patients.

Hwang et al. studied the role of CAR on stable HD patients and aimed to determine if variations in this marker were associated with mortality. In contrast to our study, they concluded that variations of CAR, rather than baseline CAR, were independently associated with long-term mortality [35]. This might be explained by the differences in the population studied as theirs was on prevalent HD patients assessing long-term mortality and ours was a study on incident HD patients and aimed at early mortality after the start of HD. Nevertheless, the value of CAR in patients with HD remains significant.

The CAR is a cheap and easily accessible marker that reflects the impact of malnutrition and inflammation on mortality in incident HD patients. With an optimal cutoff of ≥0.5, the CAR has good sensibility and specificity, with a high negative predictive value, meaning that it can safely identify patients with a low six-month mortality risk as those with CAR < 0.5. Other significant virtues of our study include a large number of patients in our cohort and the access to variables that were routinely recorded which meant that important covariates were analyzed.

Our study has some limitations to be noted. First, this was a single-center and retrospective cohort which limits the generalization of these results. We only assessed the CAR at the start of HD and did not evaluate temporal changes in CAR during follow-up, which might impact mortality. Second, we included both patients who started maintenance HD electively and urgently, which may have influenced the results as inflammation parameters could be increased due to acute illness. Third, we did not evaluate malnutrition or inflammation status by other validated methods and correlate those with CAR which would increase the value of these results. Finally, the causes of mortality were not assessed.

In conclusion, the CAR is a simple and inexpensive biomarker that can be used as an effective risk marker of early mortality in HD patients. Further multicentric and prospective studies are still required to validate this in clinical use, as should serial values of CAR and their impact on outcomes. It is crucial to identify patients with a high risk for mortality in the first months after starting HD, in order to develop and implement strategies to improve patient outcomes.

## Data Availability

Please contact author for data requests.
